# Circulating tumor cells in clinical research and monitoring patients with colorectal cancer

**DOI:** 10.18632/oncotarget.25337

**Published:** 2018-05-11

**Authors:** Claudia Burz, Vlad-Vasile Pop, Rares Buiga, Sur Daniel, Gabriel Samasca, Cornel Aldea, Iulia Lupan

**Affiliations:** ^1^ Iuliu Hatieganu University of Medicine and Pharmacy, Department Of Immunology and Allergology, Cluj-Napoca, Romania; ^2^ Ion Chiricuta Institute of Oncology, Cluj-Napoca, Romania; ^3^ Emergency Hospital for Children, Cluj-Napoca, Romania; ^4^ Babeş-Bolyai University, Department of Molecular Biology and Biotehnology, Cluj-Napoca, Romania; ^5^ Institute of Interdisciplinary Research in Bio-Nano-Sciences, Cluj-Napoca, Romania

**Keywords:** colorectal cancer, circulating tumor cells, metastasis, prognostic factor, survival

## Abstract

Colorectal cancer remains a frequent disease to which screening and target therapy exist, but despite this is still marked by a high mortality rate. Even though radical surgery may be performed in many cases, patients relapse with metastatic disease. Circulating tumor cells were incriminated for tumor recurrence, that's why vigorous research started on their field. Owning prognostic and predictive value, it was revealed their usefulness in disease monitoring. Moreover, they may serve as liquid biopsies for genetic tests in cases where tissue biopsy is contraindicated or cannot be performed. In spite of these advantages, they were not included in clinical guidelines, despite CellSearch and many other detection methods were developed to ease the identification of circulating tumor cells. This review highlights the implication of circulating tumor cells in metastasis cascade, intrinsic tumor cells mechanisms and correlations with clinical parameters along with their utility for medical practice and detection techniques.

## INTRODUCTION

Colorectal cancer (CRC) is the third most frequent cancer in men and second in women worldwide, marked by 694 000 deaths/year. In Europe it causes among all types of cancers 215 000 deaths/year [1]. More importantly, one in four patients at the diagnosis presents distant metastases and half of patients which undergo curative surgery will develop metastases. With a 5-year survival rate of 60%, CRC remains a major health problem worldwide and in areas considered in past at low risk. It is considered as a result of “westernisation”, by coping vices like heavy alcohol use, smoking, bad eating habits by consuming large amounts of red meat and sedentariness [1, 2].

Early CRC detection represents not only a favourable disease prognostic, but also a mark of an efficient treatment. In order to fulfil this condition, tumor screening is imperative. Faecal occult blood test despite being highly cost-effective, it has high false positives results [3]. Endoscopy, represented by flexible sigmoidoscopy and colonoscopy, along with double contrast barium enemas are thoroughly invasive. Modern imaging techniques such as computed tomography (CT) and magnetic resonance imaging (MRI) are highly expensive and more importantly, their application as screening methods is limited by radiation exposure. As a consequence of these issues, for early CRC diagnosis a non-invasive, valid and reasonably economical method is required [4].

Treatment of CRC varies with the disease stage. The curable one in early CRC remains surgery; however, 50% of patients develop metastases [1]. In metastatic CRC (mCRC) several therapeutic options are available, such as vascular endothelial growth factor (VEGF)-targeted therapy - Bevacizumab, anti-epidermal growth factor receptor targeted therapy - Cetuximab, Panitumumab), often associated with chemotherapy – the most active drugs being 5 Fluoro-uracil, Oxaliplatin, Irinotecan and Capecitabine. Despite the treatment progress in mCRC, the patient's prognosis is still low, with a median survival between five and 19 months [5]. Such being the case, disease monitoring and surveillance must be performed by using imaging techniques and cancer specific markers. Although improvements have been made in this field, many of these biomarkers are not used in clinical practice due to their elaborative detection or cost [6]. A series of them can be listed, from carcinoembryonic antigen (CEA), cancer antigen 19-9 (CA 19-9), microsatellite instability (MSI), V-Ki-ras2 Kirsten rat sarcoma viral oncogene homolog (KRAS), tumor protein p53 to circulating DNA, RNA and circulating tumor cells (CTCs) [7]. The last have attracted a great deal of attention to scientific research due to their roles in metastasis and possibility of being liquid biopsies as an alternative for tissue biopsies for genetic tests. Besides, they proved their importance in patients derived xenografts (PDXs) models for future drug studies, therapy monitoring and clinical parameters like overall survival (OS), progression free survival (PFS) in mCRC patients [6, 8–13]. In this context, this review endeavours to emphasize the roles, importance and detection methods of CTCs in early stage and mCRC.

## BIOLOGY OF CTCS

At early stages, from the primary tumor after epithelial-to-mesenchymal transition of primary cancer cells, CTCs begin to flow into blood stream at a rate of roughly 10^6^ cells per tumor gram [6, 14, 15]. The idea of CTCs was first implemented by Asworth in 1869. Later in 1955, Engell described the cells found in peripheral circulatory system and in the tumor blood drainage area of different types of cancer patients [4, 16]. While cells present in the blood flow are named CTCs, the ones that reach and implant in the bone marrow are called disseminated tumor cells (DTCs) [8, 10]. At this level, recent studies implemented that in certain types of cancer, including gastrointestinal malignancies, different progenitors of bone marrow stem cells represent a major key in cancer development and progression [17–19]. However, it remains unclear if these cells represent a section of the CTCs. Future research in this field must be conducted to solve the dilemma.

Adhering through cell adhesion molecules to the vascular endothelium, CTCs stand at the foundation of the biologic mechanism of metastasis in various types of cancers [20, 21]. This statement is justified by preclinical studies conducted on murine models, where human cancer cells were implanted in renal subcapsular regions and then CTCs along with metastases were detected and characterized [22].

**Figure 1:**
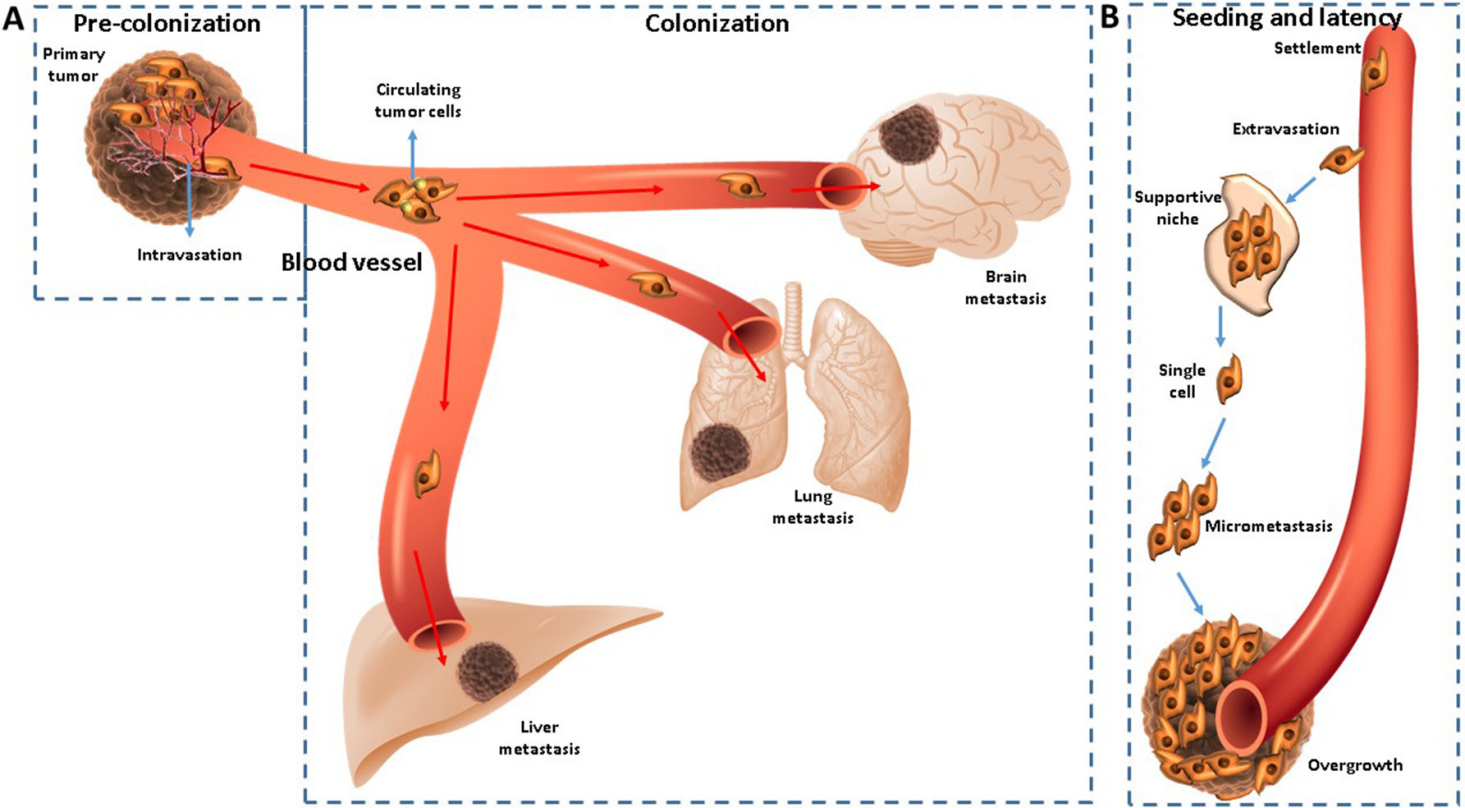
(**A**–**B**) CTCs are disseminated from primary tumor sites and a bridge for relapse or metastasis.

The process of metastasis consists of two phases: pre-colonization and colonization. The pre-colonization phase which takes part in minutes to hours on a timescale, involves intravasation of cancer cells within vasculature of the tumor, after the local invasion of them of the primary tumor. Afterwards, as single cells or platelet coated clusters, they infiltrate the circulatory system through which they pass in target organs, arresting in capillaries to start colonization. Being a laborious process, which transpires in years, colonization commences with extravasation of cancer cells, which set up resistance to host-tissue immunity in supportive niches. Subsequently, to carry out a long-standing survival, the cells as micrometastases or single units adopt a latent state. When they end latency, the process of metastasis continues with the overtaking of the target organs (Figure 1A–1B) [23].

Homing of CTCs by the viscera, as for example the liver, is not a chaotic mechanism, but in fact a controlled one, under the influence of certain natural ligands present in its vessels. The specific pathway seems to be coordinated by angiopoietin-like 6 protein. This ligand binds to E-cadherin and α [6] integrin found on the CTCs surface and thus enhances colonization of the liver [24]. Regarding the pattern of haematogenous CTCs dissemination, it was observed that the number of cells was higher in mesenteric venous blood compartments than in central one [25].

It is already known that the malignant progression requires avoidance and repression of the immune system [26]. Natural killer (NK) and cytotoxic T cells are responsible with immune surveillance over the microenvironment. Each organ consists in different specific levels of immune cells i.e. the liver, which is rich in NK. In mice, depriving the liver of pro-apoptotic NK cell-derived tumor necrosis factor-related apoptosis-inducing ligand, the process of metastasis was enhanced [23]. Reported scientific data suggests that CTCs influence innate immunity in metastatic breast, prostatic and CRC. By determining NK cytotoxicity towards cancer cells in these patients, it was observed a direct relation between CTCs and immune cells. Thus, a down regulation of the cytotoxic activity of NK was revealed in patients with high CTCs levels in peripheral blood. Moreover, this statement is supported by the decreased levels of toll-like receptors 2 and 4 that play a key role in immune response. By manipulating the immune pathways, down regulation of Ki-67, c-myc, β-catenin and over expression of CD47, CTCs seem to enhance the metastatic cascade and survive in the blood stream in a state of dormancy [27]. Their genetic profile appears to be altered, as their molecular characteristics were more concordant to metastases, than to the primary tumor [28, 29].

Immune system also plays an important role in cancer patients with severe and complicated post-operative infections such as sepsis, pneumonia, peritonitis, by enhancing metastasis and increasing the risk of death from the process. Neutrophils, which act in infections as first line of defence, are incriminated as a supporter for the metastasis progression through neutrophil extracellular traps (NETs). Formed as a response to infections, NETs consist in extruded DNA and antimicrobial proteins that have anti-bacterial, fungal and protozoal effects. However, despite the positive immune role, experimental data conducted on murine hepatic tissue revealed that CTCs are trapped in NETs and as a result, metastatic lesions increased rapidly [30]. Other cells, such as tumor-associated macrophages (TAMs) exceed their role in immune surveillance and become effectors for metastasis by producing growth factors, cytokines, chemokines as well as hormones and metabolites that hold tumor supporting effects. TAMs are derived to solid tumors as a response to chemoattractants, VEGF and endothelin-2 (ET-2), especially in hypoxic regions of growing tumors. Attracted by these factors, they interact with CTCs which inculcate TAMs to nurture tumor invasion, CTCs intravasation and their survival in the foreign microenvironment [31, 32].

Consequently, taking all these biologic mechanisms into consideration, slightly new therapeutic approaches must be taken into consideration in patients with higher number of CTCs [33].

## DETECTION AND USEFULNESS OF CTCS

Despite the large amount of CTCs released daily, they are found in low concentration in the peripheral blood. This dilemma is caused by platelets cloaks or coagulation factors that surround the CTCs, shielding them from the immune surveillance. As a result, a fraction of cells may remain undetectable [34]. Subsequently, their detection represents a challenge, mostly for developing a method of high sensibility and sensitivity [4]. So far, there were described many methods of detection such as immunocytochemistry, flow cytometry, optical fiber array scanning, reverse transcriptase polymerase chain reaction (RT-PCR), immunomagnetic separation, microchips, and others [16, 35]. From those, only the CellSearch System (Veridex LLC, Raritan, NJ) was approved by US Food and Drug Administration (FDA) in 2004 for breast, prostate and CRC, but it was not included by American Society of Clinical Oncology in clinical practice [4, 16]. With limited information on CTCs usage guidelines, didn't recommend their use only in certain situations, despite being a valid prognostic biomarker. In this matter imperative further studies must be conducted to clarify their place in clinical practice [36].

Concerning their identification, CTCs detection techniques can be realised without cell enrichment or through enrichment strategies depending on their physical or biological properties. Without enrichment, in clinical practice or on cell cultures, several methods were reported such as fiber-optic array scanning technology, density based cell mechanism combined with digital scanning microscope (AccuCyte–CyteFinder system) or Epic Platform. Enrichment based on physical properties methods isolate CTCs depending on their size, deformability, density and electric charges. In research were used many of them, like flexible microspring array, CelleSieve microfilter and the isolation by size of the epithelial tumor cells [37, 38]. Based on their biological properties, CTCs enrichment strategies are divided in two methods. Positive enrichment techniques capture CTCs and release normal cells, i.e. Epithelial cells adhesion molecule (EpCAM) used in CellSearch, immunomagnetic beads coated with antibodies like cytokeratin 20 (CK20). The negative enrichment, with an opposite mechanism of the first one, uses the negative marker CD45 or other CDs for detection [15, 38, 39]. However, some changes in the CTCs molecular biology interfere in the detection process. It was revealed using *in vitro* evidence, that in mCRC patients, CellSearch may be prevented in detection of CTCs by the Bevacizumab therapy. Its sensibility seems to decrease after long exposure of cells to the VEGF-antibody, due to the low expression of EpCAM 40 kDa isoform and increase expression of the isoform EpCAM 42kDa [40].

Often, for a more exact detection, two or more methods were combined for a more specific counting. CellSearch and AdnaTest which uses an RT-PCR method to detect specific cells from the EpCAM enriched fraction, was used in mCRC and was found superior to using one single method [41].

Besides, new technologies are imagined and used *in vitro* or *in vivo* in different types of cancers. In the last fifteen years, several methods were used for CTCs detection and analysis [42]. Many scientists tried to invent and optimize their method to add it in clinical protocols (Table 1).

**Table 1:** Different methods for CTCs detection and analysis

Detection method	Reference
Biomimetic lipid coated microfluids	[43]
Celsee device microfluidic chip-based	[44]
Micromagnet-integrated microfluidic screening system	[45]
Inkjet-printed microscale magnetic structure on glass slides	[46]
Electrical detection method using graphene nanoplates	[47]
Immunomagnetic negative enrichment and fluorescence-activated cell sorting	[48]
Size-based isolation with a novel filter device (FMSA)	[38, 49]
Nanostructured polystyrene well plates	[50]
Filter separation and 5-aminolevulinic acid (5-ALA)	[51]
Multiplex-PCR	[38, 52]
Micro-Raman microscopic	[53]
Biocompatible TiO2 nanoparticle-based cell immunoassay	[54]
DEPArray dielectrophoresis-based platform	[38, 55]
Microfluidic bead-based multienzyme-nanoparticle amplification	[56]
Hybrid polydimethylsiloxane microfluidic device	[57]
Quantum-dot-labelled magnetic immunoassay	[58]
Electrospun TiO2 nanofiber-based cell capture assay	[59]
ScreenCell Cyto	[60]
Epispot assay	[38, 61]
CELLection Epithelial Enrich system	[62]
High-throughput microsampling unit (HTMSU)	[63]

Despite their variety, it remains imperative to select or generate a sensible and specific detection method. Afterwards, it must be verified on a suitable number of patients to prove CTCs importance and clinical utility as markers for medical practice.

Besides their molecular characteristics, the usefulness of peripheral blood CTCs in clinical implication was seen to have a great importance in early stage and metastatic cancer. In non-metastatic CRC patients, CTCs detected preoperative represent a valid prognostic factor for cancer progression and survival [64]. In patients that underwent surgical resection of the primary tumor, CTCs revealed an increased risk of postoperative metastasis [65]. Their persistent presence after curative resection was associated with poor prognosis and relapse free survival (RFS) [66].

In mCRC data from relevant studies unveiled that they are predictive markers for chemotherapy through discerning the potential metastases prematurely, choosing patients resistant to chemotherapy and ascertaining clinical outcomes. Being a prognostic marker, in particular cases of mCRC, where levels of CEA and other markers were found not measurable, CTCs fulfilled a better disease monitoring [6]. More important, CTCs high levels were associated with clinical outcome parameters like worse progression free survival (PFS) and overall survival (OS) in CRC patients [6, 66–68]. As a further matter, higher level of CTCs was correlated with tumor relapse due to their conversion in cancer stem cells that start recurrence [69].

Epithelial to mesenchymal transition (EMT) is a process which CTCs gain to enable metastasis. In mCRC patients, by examining the CTCs-microRNA (mRNA) expression of EMT different transcripts implicated in cell migration, it was observed a correlation with OS and PFS [70]. Other studies conducted on healthy donors and mCRC patients, revealed that mRNA molecular characterization of CTCs is possible in order to attempt further individualized treatment [71].

Furthermore, CTCs were revealed to diagnosis liver metastases (LM). Using flow cytometry to detect cellular subpopulations of CTCs in mCRC with LM and non-metastatic CRC, it was observed that CD133, CD54 and CD44 were higher in mCRC-LM patients, proving the strong association between these cellular subpopulations and LM. Untimely and right LM diagnosis allows the option of performing a liver-targeted therapy to improve survival. By merging modern abdominal imaging with CEA, CA 19-9 and CD differentiation of CTCs, it was unveiled an increased sensitivity and specificity in detection the LM. Thus, CTCs may play a role in this matter as a predictive and auxiliary diagnosis marker [72].

To strengthen the argument of auxiliary markers, there were conducted a series of studies on chemotherapy treated mCRC. Each study detected patient's CTCs at different points in their evolution and concluded the correlations between CTCs and imaging response to therapy, PFS and OS (Table 2).

**Table 2:** Comparison between different studies on CTCs levels and imaging response to therapy, PFS and OS

Reference & No. patients	Chemotherapy	CTCs evaluation	Response to Imaging and CTCs levels					PFS	OS
Cohen SJ. 430 P [73]	Heterogeneous	CellSearch System	RECIST 3–5 weeks	< 3		> 3		7.2 M (95% CI, 6.7–7.9 M)	15.5 M (95% CI, 14.0–18.4 M)
				No.	%	No.	%		
			RR (SD, PR, CR)	228	93	18	7		
			PD	54	73	20	27		
Tol J. 467 P [74]	XELOX +Bevacizumab +/− Cetuximab	CellSearch System	RECIST 1–2 weeks	High		Low		10.0 M (95% CI, 8.8–11.2 M) - low 3.9 M (95% CI, 1.7–5.4M) - high	20.0 M (95% CI, 17.8–21.4 M) - low 6.3 M (95% CI, 3.3–10.5 M) - high
				No.	%	No.	%		
			Response	2	11	115	40		
			SD	12	67	158	55		
			PD	4	22	16	5		
Matsusaka S. 61 P [75]	FOLFOX4 +/−Bevacizumab	CellSearch System	RECIST 8–12 weeks	< 3		> 3		1.9 M (95% CI, 0.5–3.3 M) > 3 9.1 M (95% CI, 7.6–10.7 M) < 3	4.1 M (95% CI, 0–11.7 M) > 3 29.1 M (95% CI, 20.3–38.0 M) < 3
				No.		No.			
			RR (SD, PR, CR)	52		1			
			PD	4		3			
Sastre J. 180 P [76]	XELOX +Bevacizumab	CellSearch System	RECIST 8–12 weeks	< 3		> 3		10.8 M (95% CI, 9.7–12.5 M) < 3 7.5 M (95% CI, 4.0–9.9M) > 3	25 M (95% CI, 20.0–28.3) < 3 16.1M (95% CI, 9.2–26.0 M) > 3
				%		%			
				53,2		26,1			
Alburqueque A. 33 P [77]	Heterogeneous	Immunomagnetic enrichment with BM7 and VU1D9 antibodies	n/a					181 days (95% CI, 146.9–215.1 - positive CTCs) 329.0 days (95% CI, 299.6–358.4) - no CTCs	n/a
Barbazan J. 50 P [78]	Heterogeneous	Multimarker CTCs detection panel	RECIST 4-W	High		Low		12.1 M (95% CI, 9.7–14.4) - low 7.3 M (95% CI, 4.4–10.2) - high	23.6 M (95% CI, 19,9–27,3) - low 12.4 M (95% CI, 7,3–17,6) - high
				No.	%	No.	%		
				38	76	12	24		

Abbreviations: CR, Complete Response CT, computed tomography; CTCs, circulating tumour cells; M, Months; No., number of; OS, overall survival; P, patients; PD Progressive Disease, PFS, progression free survival; PR, Partial Response, RECIST, Response Evaluation Criteria in Solid Tumours; RR, Response Rate; SD, Stable Disease; W, weeks.

However, there are a series of discordances between patient's treatments, time of blood samples collection, CTCs levels reference value, detection methods and imaging methods used for disease staging. Even if it's clear that CTCs reflects therapy response, the differences must be settled in order to strengthen their importance as useful markers for clinical practice.

Consequently, in early stage CRC CTCs detection is correlated with cancer progression and poor prognosis, whereas in mCRC the biomarker high levels indicate disease progression, along with on overall poor outcome [64].

Besides their roles mentioned until this point, their implication as auxiliary markers represents more than meets the eye. CTCs were proposed to be used as liquid biopsies as an alternative for tissue biopsies for precision medicine or in PDXs models because of their effortless collection and possibility of tracking disease evolution at any step [9, 13, 79].

Similar to CTCs, circulating cell-free DNA (cfDNA) has attracted a great deal of attention in biomedical research. Detected in the cell-free portion of whole blood, cfDNA was first reported in 1948 by Mandel and Metais and since, it remains a promising area of research in many medical disciplines. Exercise, trauma, myocardial infarction, stroke, end-stage kidney failure represent some of the situations that increase the levels cfDNA. In cancer patients, a minor portion of cfDNA is represented by circulating tumor DNA (ctDNA) from tumor shedding. In order to guide targeted therapy, molecular analysis of tumor tissue is imperative to detect the gene mutations. This process is sometimes hampered by heterogeneity of the primary tumor and metastases [80]. Moreover, the biopsy itself does not lack of limitations such as clinical complications, costs and invasiveness. Taking these arguments into account, ctDNA released from necrosis of cancer cells represents an alternative to the tissue sampling because it can be collected at any time to observe tumor dynamics and mutations, amplifications, rearrangements. Due to an about 2 hour's half time ctDNA enables a better evaluation of tumor dynamics over imaging techniques or conventional biomarkers [81]. Moreover, it can bring essential information about tumor burden, minimal residual disease, the mechanisms of molecular drug resistance and heterogeneity, as well as disease monitoring [81–85].

Both of the biomarkers, CTCs and ctDNA, reflect tumor spatial and temporal heterogeneity, evolution and mutations. In addition, they represent non invasive biopsy methods with a high specificity. Despite this beneficial values, each of these liquid biopsies present a series of limitations and strengths. Regarding the phenotype, genotype, cell cultures and PDXs models of tumor, CTCs represents the most suitable biomarker. From the opposed point of view, ctDNA proves its utility in monitoring the treatment response and relapse [38]. Thus, both are interdependent as liquid biopsies, but present a series of others drawbacks and advantages that define CTCs and ctDNA suitable in different directions (Table 3) [80].

**Table 3:** Advantages and disadvantages of ctDNA and CTC [38, 80]

	CTC	ctDNA
**Advantages**	-Appraise expression of proteins -*Ex vivo* functional studies -*In vitro* analyses for evaluation of treatment sensitivity -Useful for optimizing therapy selection by DNA, RNA and proteins analysis of cells -Early cancer detection -Establishing carcinoma origin	-Increased genome amount per unit volume which is more sensitive -Monitoring disease burden -Monitoring therapy response and relapse by gathering molecular information -Possibility to elucidate mechanisms of drug-resistance
**Disadvantages**	-False-positive results by detecting benign circulating epithelial cells in benign inflammatory disease (Crohn disease) -Heterogeneity of the population of CTC	-Without expression of proteins -Impossibility to assess functional studies -Limited molecular profiling -False-positive results of insignificant molecular mutations due to method high sensitivity -variable levels of ctDNA in persons -In early cancer stages it may be hard to detect

However, overall mutations detected in CTCs and ctDNA like KRAS, BRAF and PIK3CA were virtually in concordance [28]. In clinical practice, KRAS was revealed to have major implication in CRC patients. Its mutation itself is a negative criterion for treatment with targeted anti-EGFR monoclonal antibodies like Cetuximab or Panitimumab. To set up the targeted treatment, KRAS mutation must be assessed from tumor tissue. Due to the difficulty of obtaining an appropriate sample for KRAS genotyping, other alternatives were taken into consideration, like molecular characterization of CTCs. Specific methods, as droplet digital PCR or performing KRAS kits by quantitative PCR, nested Allele-Specific Blocker PCR or wild-type blocking PCR, revealed that is possible to assess KRAS mutation in CTCs from peripheral blood [86–89]. The minimal invasive method could be implemented in clinical practice because of the insight information obtained.

In addition, a stronger prognosis indicator of CTCs was seen through the analysis of programmed death-ligand (PD-L1) in CRC patients undergoing treatment. Its expression was quantified at cells membrane, cytoplasm and nucleus. From those, the nucleus high expression was correlated with shorter patient's survival durations [90]. Another recent marker for CTCs is represented by Plastin-3 (PLS3) encoded by a gene on a chromosome Xq23. With a role in EMT induction and invasiveness, PLS3 protein high expression was correlated with poor prognosis of patients with CRC and with a high rate of metastasis [91].

Altogether, the relevance of CTC determination relies in the fact that they represent prognostic and predictive markers for CRC patient. For their role, significance and correlations with clinical outcome, CTCs tend to make the transition from scientific research to clinical guidelines implementation.

## CONCLUSIONS

CTCs remain a subject of major interest for scientific research by standing at the base of metastasis cascade. Moreover, as revealed by many studies, they could represent early prognostic and predictive markers in early and mCRC, revealing crucial information on disease monitoring. In addition, CTCs might serve as non invasive liquid biopsies to test different genetic mutations, like VEGF, KRAS, allowing initiation with targeted therapy such as Bevacizumab, Cetuximab or Panitumumab. The shortage of consensus, due to many differences in detection methods, time of detection and reference value recognize a limited use of CTCs in CRC management.
